# What are the microbial burden and potential pathogens on hospital surfaces before and after cleaning?

**DOI:** 10.3389/fmicb.2026.1835355

**Published:** 2026-07-15

**Authors:** Clément Legeay, Carole Lemarié, Matthieu Eveillard

**Affiliations:** 1 Infection Control and Prevention Unit, CHU Angers, Angers, France; 2 Laboratoire de Bactériologie, Département de Biologie des Agents Infectieux, CHU Angers, Angers, France

**Keywords:** bioburden, environmental contamination, healthcare-associated infections, hospital cleaning, infection prevention and control

## Abstract

Healthcare-associated infections (HCAIs) remain a significant challenge in hospitals, with environmental contamination increasingly recognized as a contributing factor. However, the relationship between surface microbial burden, pathogen presence, and the effectiveness of cleaning interventions remains incompletely understood. This study aimed to describe the microbial contamination of hospital surfaces before and after cleaning, evaluate the impact of an educational intervention targeting environmental services (EVS) staff, and assess the relevance of two cleanliness thresholds (4 CFU/cm^2^ and 2.5 CFU/cm^2^) for predicting pathogen presence. A prospective study was conducted over a 5-month period across 10 wards in a 1,600-bed university hospital. Surface contamination was assessed weekly using contact agar plates to sample 15 high-touch surfaces and floors in randomly selected rooms, both before and after routine cleaning. Cleaning performance was concurrently monitored using fluorescent markers. In addition, a 2-day training program was delivered to 50% of EVS staff. Microbial burden (CFU/cm^2^), the prevalence of contaminated surfaces, and the presence of clinically relevant pathogens were analyzed. A total of 736 samples were collected. Cleaning significantly reduced the overall microbial burden (mean 1.05 vs. 0.75 CFU/cm^2^, *p* < 0.001) and pathogen prevalence (19.0% vs. 13.1%, *p* = 0.03). Floors were the most frequently contaminated surfaces, whereas high-touch surfaces exhibited lower contamination rates. Pathogen presence was strongly associated with higher bioburden: 53% vs. 10% for surfaces with >4 CFU/cm^2^ compared with those with ≤ 4 CFU/cm^2^, and 56.3% vs. 5.9% for surfaces with >2.5 CFU/cm^2^ compared with those with ≤ 2.5 CFU/cm^2^ (*p* < 0.0001 for both). The educational intervention significantly improved cleaning compliance as assessed using fluorescent markers (40.3% vs. 62.8%, *p* < 0.001), though reductions in overall bioburden were not statistically significant. In this study, we found that a substantial proportion of hospital surfaces remained contaminated with pathogens even after routine cleaning. Lower microbial thresholds, particularly a threshold of 2.5 CFU/cm^2^, may be relevant indicators of cleanliness for high-touch surfaces. Targeted training improved cleaning practices and showed a trend toward bioburden reduction, highlighting the importance of structured interventions in HCAI prevention strategies.

## Introduction

Healthcare-associated infections (HCAIs) affect 4.3 million patients annually in Europe (European Centre for Disease Prevention and Control., 2024). In French hospitals, the prevalence of HCAIs is 6.4%, with a significant proportion being associated with antimicrobial-resistant microorganisms (European Centre for Disease Prevention and Control., 2024).

Hospital surfaces have traditionally been considered minor reservoirs for pathogens, and their role in HCAIs remains debated. However, several studies have demonstrated an increased risk of pathogen acquisition from prior room occupants (Mitchell et al., 2023). Furthermore, improved environmental hygiene has been associated with reduced HCAI rates (Peters et al., 2022; Mitchell et al., 2019).

Surface cleaning in hospitals temporarily reduces bioburden. However, it is often performed by underpaid, undervalued environmental services (EVS) staff with little to no training (Gon et al., 2024). Up to 50% of hospital surfaces remain contaminated after cleaning (Carling et al., 2008). Improving hospital cleaning through structured interventions—such as staff training, promoting an institutional safety climate, monitoring, and feedback—can enhance cleanliness and potentially reduce HCAI rates (Trajtman et al., 2013; Martin et al., 2019; Hayden et al., 2006; Hamed et al., 2024).

Several tools are available to assess cleanliness. Visual inspection is simple and inexpensive but lacks accuracy (Chen et al., 2021). Fluorescent markers and ATP bioluminescence allow for rapid feedback to cleaning personnel (Carling, 2013), but they do not provide information on microbial contamination. Microbial sampling also has limitations, as it is costly, labor-intensive, and results are delayed.

Several studies have described the microbial burden on hospital surfaces. A recent study conducted across four hospitals in England found that half of the sampled surfaces yielded >10^3^ CFU/cm^2^ (Watson et al., 2023). Moreover, clinically relevant pathogens are frequently detected on high-touch hospital surfaces (Jinadatha et al., 2024).

Currently, there is no standardized threshold to define a “clean” surface in hospital rooms. A threshold of 100 CFU per sampled surface (4 CFU/cm^2^ on a 25 cm^2^ contact plate) has been associated with a higher probability of pathogen detection (Koff et al., 2024). A threshold of 2.5 CFU/cm^2^ for total aerobic colony counts and a threshold of <1 CFU/cm^2^ for pathogens have also been proposed as a benchmark for hospital surface cleanliness (Dancer, 2023).

In this study, we aimed to describe surface bioburden in hospital rooms before and after cleaning, evaluate the impact of a training intervention on cleaning performance, and assess the association between pathogen presence and two microbial thresholds (4 CFU/cm^2^ and 2.5 CFU/cm^2^).

## Methods

### Setting

This study was conducted in a single building of a 1,600-bed university hospital between November 2019 and March 2020, covering five surgical and five medical wards. Thirty EVS staff members were involved in room cleaning in the building.

### Cleaning monitoring

Cleaning performance was assessed using two complementary methods in randomly selected patient rooms.

#### Fluorescent markers

Cleaning was monitored using fluorescent markers (DAZO – Ecolab^®^). The fluorescent marker was applied to 15 frequent touch points and to the floor in patient rooms and toilets in the morning (1–2 h after daily cleaning). Cleaning performance was assessed based on the removal of fluorescence after 24 h (i.e., evaluated 1–2 h after daily cleaning). EVS staff members were blinded to the marked rooms. These audits were conducted from Monday to Thursday, with two randomly selected rooms marked each day. Data collection was structured into three phases: baseline (September 2019–January 2020), early intervention (January–March 2020), and late intervention (June–December 2020).

#### Surface sampling

Surface contamination was assessed weekly in one randomly selected room of each group between November 2019 and March 2020. Trypticase soja (for bacteria) and Sabouraud (for fungi) contact plates were used on 15 high-touch surfaces and the floor before cleaning (1 h prior) and after cleaning (1 to 2 h later). EVS staff were blinded to the sampled rooms.

We assessed the prevalence of surfaces with values of >4 CFU/cm^2^ or < 4 CFU/cm^2^; < 2.5 CFU/cm^2^ or < 2.5 CFU/cm^2^; and the average bioburden (expressed in CFU/cm^2^). Pathogens included *Enterobacterales, Staphylococcus aureus, Pseudomonas aeruginosa, Acinetobacter baumannii, Enterococcus* species, *Candida* species, and *Aspergillus fumigatus*. Microbial identification was performed using MALDI-TOF mass spectrometry (Vitek MS, bioMérieux, France).

Oral consent was obtained from patients before marking or sampling the rooms. Marking and sampling were performed by five different trained infection prevention and control (IPC) members, according to a standardized protocol (including photos of the different types of rooms where surfaces to mark/sample were identified).

If a randomly selected room for marking/sampling was vacant, an adjacent occupied room was selected for monitoring.

### Cleaning procedures

Cleaning procedures remained unchanged throughout the study, with personnel using a quaternary ammonium-based detergent/disinfectant for both cleaning and disinfection.

EVS staff members started by cleaning the furniture in the patient room and toilets, followed by floor cleaning of the room and toilets. This daily cleaning took place in the morning; one EVS staff member was assigned to 15–20 rooms. Following patient discharge, two EVS staff members cleaned the room using the same procedure. Rooms with infectious precautions (multidrug-resistant carriers, *Clostridioides difficile*, viral infections, and other transmissible infections) were cleaned last.

### Cleaning improvement–training program

In January 2020, a 2-day training program was conducted by two members of the IPC team. Two sessions, held 1 week apart, were scheduled to maintain small groups (7–8 trainees). In total, 15 EVS staff members (50% of the targeted professionals) attended the program.

Day 1 focused on theory regarding environmental contamination and cleaning products. To raise awareness regarding bioburden, participants were given a contact agar plate to sample any surface of their choice in a patient room, and the plate was evaluated for microbial growth 24 h later. On day 2, several practical exercises were performed in a simulated environment to develop technical skills. A major educational objective was to give the activity a strong message and emphasize its importance in HCAI prevention.

### Statistics

Pre- and post-cleaning samples were analyzed as independent observations, as surfaces were not individually paired. Categorical variables were compared using the chi-square test (with Yates' correction when applicable) or Fisher's exact test, as appropriate. Continuous variables were compared using the Mann–Whitney U-test. A *p*-*value* of < 0.05 was considered statistically significant.

## Results

### Microbial burden on hospital surfaces

A total of 736 samples were analyzed (399 pre-cleaning and 337 post-cleaning).

Parameters used to assess cleanliness improved after cleaning (Table 1). Nonetheless, we found that 13.1% of surfaces still displayed at least one clinically relevant pathogen after cleaning.

**Table 1:** Hospital surfaces contamination before and after cleaning.

Surface contamination	Before cleaning	After cleaning	*p*
Average colony count per plate	26.16 (1.05 CFU/cm^2^)	18.64 (0.75 CFU/cm^2^)	< 0.001
Surfaces with >4 CFU/cm^2^	28.8%	13.9%	< 0.001
Surfaces with >2.5 CFU/cm^2^	29.8%	14.2%	< 0.001
Pathogen prevalence	19.0%	13.1%	0.03

The microorganisms identified on hospital surfaces after cleaning are shown in Table 2.

**Table 2:** Prevalence of microorganisms on hospital rooms' surfaces after cleaning.

Type of microorganisms	*N* (%)
Microorganisms not considered major pathogens	
- Coagulase-negative staphylococci	128 (38.0)
- Bacillus spp	37 (11.0)
- Others	160 (47.5)
Pathogens	
- *Enterobacterales*	13 (3.9)
- *Staphylococcus aureus*	16 (4.7)
- Non-fermenting gram-negative bacilli	5 (1.5)
- *Enterococcus* spp	11 (3.3)
- Fungi^*^	13 (4.2)
° *Candida* spp	11 (3.6)
° *Aspergillus fumigatus*	2 (0.6)

^*^307 samples were collected for fungi presence.

Room and bathroom floors were frequently still contaminated after cleaning. Surfaces in contact with hands, considered “critical surfaces,” were much less frequently contaminated (Supplementary material).

### Impact of total aerobic colony count thresholds on pathogen detection

On surfaces with >4 CFU/cm^2^, we found a 53% prevalence of pathogens, compared with 10% on surfaces with < 4 CFU/cm^2^ (*p* < 0.0001).

Concurrently, the proportion of surfaces with values of >2.5 CFU/cm^2^ was associated with a higher pathogen prevalence than those with values of < 2.5 CFU/cm^2^ (56.3% vs. 5.9%, respectively, *p* < 0.0001).

### Impact of educational intervention on hospital surface contamination

The educational intervention significantly improved cleaning compliance, as assessed by fluorescent markers (40.3% of markers removed before the intervention vs. 62.8% after the intervention, *p* < 0.001).

We observed a reduction in global bioburden on hospital surfaces after cleaning, both in the prevalence of surfaces displaying >4 CFU/cm^2^ and >2.5 CFU/cm^2^ and in the average CFU per plate, but these reductions were not statistically significant (Table 3). Pathogen prevalence was roughly the same.

**Table 3:** Global microbial burden on hospital surfaces after cleaning, before and after an educational intervention to improve cleaning.

Surface contamination	Before intervention	After intervention	*p*
Average colony count per plate	20.4 (0.82 CFU/cm^2^)	16.9 (0.68 CFU/cm^2^)	0.12
Samples with ≥4 CFU/cm^2^	29/172 (16.9%)	18/165 (10.9%)	0.16
Samples with >2.5 CFU/cm^2^	30/172 (17.4%)	18/165 (10.9%)	0.12
Pathogen prevalence	23/172 (13.4%)	21/165 (12.7%)	0.99

## Discussion

In this 5-month study, which included more than 700 surface samples, we found that, although cleaning improved overall surface cleanliness, 13.1% of surfaces remained contaminated with clinically relevant pathogens after routine cleaning.

*Staphylococcus aureus* and *Enterobacterales* were among the most frequently detected pathogens. These microorganisms are known to harbor antimicrobial resistance mechanisms and are commonly found in the hospital environment (Mody et al., 2019; Shams et al., 2016). The presence of fungi is rarely assessed as part of the overall microbial burden. We found that 4.2% of surfaces exhibited *Candida* and/or *Aspergillus fumigatus* after cleaning. Notably, *Candida parapsilosis* was detected on 82% of surfaces with *Candida* spp. Hospital surfaces are increasingly recognized as a source of invasive candidiasis caused by *Candida parapsilosis* (De-la-Pinta et al., 2026). Further studies assessing the prevalence of this pathogen in hospital settings and its resilience to current cleaning strategies should be conducted.

The presence of pathogens was associated with total colony counts. Notably, on surfaces with less than 2.5 CFU/cm^2^, pathogens were rare (<6%). The correlation between total microbial burden and pathogen presence remains controversial. Some studies have not identified such a correlation (Widmer et al., 2019; Shams et al., 2016). On the contrary, in a recent study conducted in nine Canadian hospitals, pathogens were recorded in 23% of samples taken from anesthesia machines with a total colony count of >100 CFU, compared with 3% in those with <100 CFU (Dexter et al., 2024). The same team confirmed a 100 CFU threshold in a recently published study of high-touch surface samples from intensive care units (pathogen present in 40% and 14% of samples with >100 CFU and < 100 CFU, respectively (Koff et al., 2024). Another recent study assessing toilet microbial contamination found that over 80% of pathogens were associated with a bioburden >2.5 CFU/cm^2^ (Aumeran et al., 2025). This threshold of 2.5 CFU/cm^2^ could be relevant for monitoring “critical” surfaces (i.e., those in contact with hands), as previously suggested (Dancer, 2014).

Hospital environmental cleaning is a crucial yet often overlooked component of HCAI prevention. There is a need for increased recognition and improvement of this activity. In this study, before the educational intervention, we found that approximately 40% of fluorescent markers on hospital surfaces were adequately removed after cleaning. These results are consistent with previous publications (Carling and Bartley, 2010; Boyce et al., 2010). Our educational intervention was associated with increased cleaning compliance, but the rates were still insufficient. It might be due to the fact that our intervention reached only 50% of the target audience. Availability of EVS for the teaching program is challenging. These teams suffer from absenteeism. We recently evaluated in an unpublished study that up to 5 EVS staff members were on sick leave among 14 workers over several weeks (unpublished data). Teaching interventions alone might be insufficient for these highly exposed workers. Work conditions need to be addressed, along with improved pay scales, communication, and management strategies (Dancer, 2023). A limitation of the assessment of our teaching intervention was the before–after design. A more robust evaluation could be conducted with randomization of EVS staff to attend or not attend the teaching program in an intervention/control study. With a high level of absenteeism and a high risk for cross-contamination from trained personnel to those in the “control” group, we have decided not to pursue an intervention/control evaluation but rather to assess the global impact of a partial teaching program.

Our study showed that improved hospital cleaning was associated with a non-significant bioburden reduction. The correlation between fluorescent dye removal and bioburden reduction is still unclear (Carling, 2013). Wiping a surface with a microfiber cloth (as used in this study) may remove the fluorescent marker, but if the mechanical action is not sufficient, microorganisms can persist. The discrepancy we observed between a significant improvement in fluorescent removal but a non-significant reduction in microbial burden also raises the question of the actual effectiveness of the detergent/disinfectant used for hospital cleaning. Nevertheless, this post-intervention improvement is promising. Further studies should be conducted to assess the impact of educational interventions for EVS, associated with audits and immediate feedback, to confirm the previously encouraging results (Mitchell et al., 2019).

Fluorescent markers, though their correlation with microbial load is debated (Dewangan and Gaikwad, 2020; Hung et al., 2018; Fattorini et al., 2016), remain a practical tool for assessing cleaning performance. Our study was not designed to compare fluorescent marker assessment and microbial sampling, but both showed improvement after intervention. Fluorescent markers offer immediate feedback to EVS staff and can help locate poorly cleaned surfaces for improvement (Parry et al., 2022). Recently, a study conducted in an intensive care unit found that fluorescent marking and feedback were associated with a reduction in cleaning failures and a non-significant reduction in multidrug-resistant organism infection or colonization (Ziegler et al., 2022).

This study has several limitations. Sampling was performed 1 to 2 h after cleaning, which may have allowed for recontamination and could have led to an underestimation of cleaning efficacy. We did not collect patient-level microbiological data and therefore could not assess the relationship between patient colonization or infection and environmental contamination. Although EVS staff members were blinded to marked rooms, they were aware of the audit; therefore, it is possible that the Hawthorne effect contributed to improved cleaning, as previously described (Guerrero et al., 2013). Due to the COVID-19 pandemic that impacted our hospital in March 2020, we did not conduct a long-term assessment of our educational intervention.

Our study shows that 13.1% of surfaces remained contaminated with pathogens within 1 to 2 h post-cleaning in medical/surgical wards. *Enterobacterales* and *Staphylococcus aureus* were frequently found pathogens on hospital surfaces, highlighting the need to improve cleaning and hand hygiene to prevent HCAIs and cross-transmission. A threshold of 2.5 CFU/cm^2^ appears relevant for assessing cleanliness on “high-touch” hospital surfaces.

## Data Availability

The raw data supporting the conclusions of this article will be made available by the authors, without undue reservation.
