# Supplementary material for: What are the microbial burden and potential pathogens on hospital surfaces before and after cleaning?
Source: Front Microbiol. 2026 Jul 15;17:1835355. doi: 10.3389/fmicb.2026.1835355 (PMC13415349; doi:10.3389/fmicb.2026.1835355)
Supplement: Supplementary file 1 [file Table_1.docx]

Surfaces displaying > 4 CFU/cm² or pathogens after cleaning.

|  | > 4CFU/cm² | Pathogens prevalence |
| --- | --- | --- |
| Door handle | 8.3% | 0% |
| Light switch | 4.2% | 8.3% |
| Table | 8.3% | 4.2% |
| Armchair | 17.4% | 8.7% |
| Bedside table | 8.3% | 4.2% |
| Overbed table | 0% | 4.2% |
| Room floor | 52.2% | 43.5% |
| Bed rail | 4.3% | 0% |
| Bed remote control | 8.3% | 12.5% |
| Doorbell | 13.0% | 4.3% |
| Bathroom floor | 85.0% | 80.0% |
| Faucet | 10.0% | 10.0% |
| WC grab bar | 10.5% | 10.5% |
| Toilet flush button | 0% | 4.8% |
| Toilet bowl | 0% | 5.0% |
